# Recycling of Coal Fly Ash for the Fabrication of Porous Mullite/Alumina Composites

**DOI:** 10.3390/ma7085982

**Published:** 2014-08-19

**Authors:** Kyu H. Kim, Seog Y. Yoon, Hong C. Park

**Affiliations:** School of Materials Science and Engineering, Pusan National University, Pusan 609-735, Korea; E-Mails: kgh31202@gmail.com (K.H.K.); syy3@pusan.ac.kr (S.Y.Y.)

**Keywords:** coal fly ash, mullite/alumina, camphene, freeze casting, pore channels, compressive strength

## Abstract

Coal fly ash with the addition of Al_2_O_3_ was recycled to produce mullite/alumina composites and the camphene-based freeze casting technique was processed to develop a controlled porous structure with improved mechanical strength. Many rod-shaped mullite crystals, formed by the mullitization of coal fly ash in the presence of enough silicate, melt. After sintering at 1300–1500 °C with the initial solid loadings of 30–50 wt.%, interconnected macro-sized pore channels with nearly circular-shaped cross-sections developed along the macroscopic solidification direction of camphene solvent used in freeze casting and a few micron-sized pores formed in the walls of the pore channels. The macro-pore size of the mullite/alumina composites was in the range 20–25 μm, 18–20 μm and 15–17 μm with reverse dependence on the sintering temperature at 30, 40 and 50 wt.% solid loading, respectively. By increasing initial solid loading and the sintering temperature, the sintered porosity was reduced from 79.8% to 31.2%, resulting in an increase in the compressive strength from 8.2 to 80.4 MPa.

## 1. Introduction

The utilization of industrial waste to produce highly valuable products attracts a great deal of attention from society in general, and scientists in particular. The recycling of a vast amount of coal fly ash generated in thermal power plants is one of the serious problems which must be solved from the viewpoint of environmental protection, from insufficient filling-up and resource re-creation. The annual production of coal ash in South Korea is about 8.6 million tons (2012) [1]. Coal fly ash consists of fine inorganic particles containing some noncombustible residues and its main components are SiO_2_ and Al_2_O_3_ . Therefore, the chemical composition of coal fly ash enables the synthesis of mullite (3Al_2_O_3_·2SiO_2_). In this case, an appropriate amount of Al_2_O_3_ is commonly added to the coal fly ash to increase the molar ratio of Al_2_O_3_/SiO_2_ because coal fly ash has deficient alumina content to the stoichiometric 3/2-mullite. Mullite is widely used as advanced structural ceramic because of its high strength, good thermal and chemical stability [2].

Porous ceramic composites are applied to gas/liquid filters, catalyst carriers, the removal of diesel particulates, sound-absorbing materials, and electrodes in fuel cells [3,4,5]. Almost all applications of porous ceramics require both high porosity and good mechanical strength. Since the mechanical strength is in inverse proportion to porosity, processing designs for achieving higher mechanical strength with a smaller decrease in porosity are desirable. Several processing routes which involve direct foaming, replica and sacrificial template, are proposed to fabricate porous ceramics [6]. Because each of these techniques has its own drawbacks as well as merits, it is difficult to simultaneously satisfy structural and mechanical requirements. Freeze casting is a potential shaping method to prepare porous ceramics with controlled pore structure [7,8,9]. This process consists of preparing a stable ceramic suspension that is poured into a mold, freezing a liquid suspension, and sublimating the solidified dispersing medium. The resulting pore morphology obtained from this method strongly depends on the selected solvent and its freezing conditions including freezing direction, freezing rate and temperature [10,11,12].

The objective of this work is to facilitate the recycling of coal fly ash for high value added resources and to produce porous mullite/alumina composites with a tailored porous structure by freeze casting. In addition, the influence of processing variables (solids loading and sintering temperature) on the microstructure and mechanical strength of resulting products has been examined. In this study, camphene was selected as a dispersing solvent. An appropriate Al_2_O_3_was added to coal fly ash to produce mullite/alumina composites.

## 2. Results and Discussion

**Figure 1:**
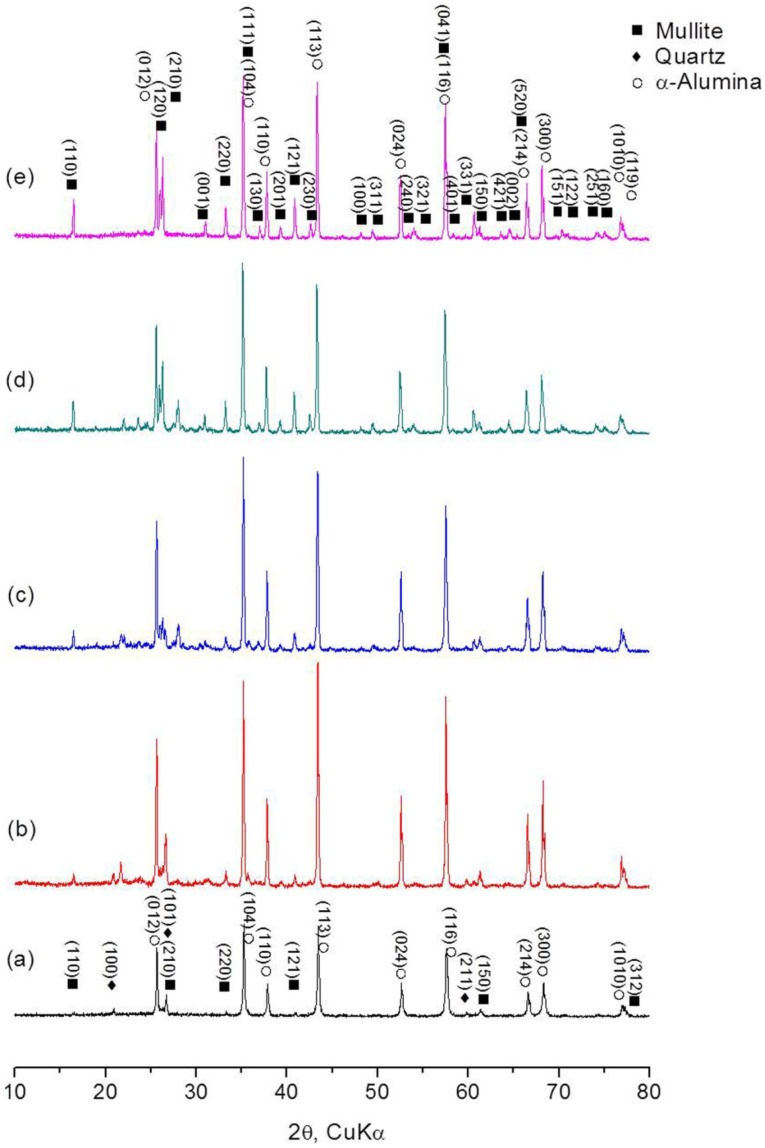
XRD patterns of (**a**) mixed batch powder of coal fly ash and Al_2_O_3_, and freeze cast bodies sintered at (**b**) 1000 °C; (**c**) 1200 °C; (**d**) 1300 °C and (**e**) 1400 °C.

Figure 1 shows XRD patterns of the freeze cast materials sintered at 1000–1400 °C, together with mixed batch powder of coal fly ash and Al_2_O_3_. The characteristic peaks of mullite and corundum (α-Al_2_O_3_) were confirmed in all samples and the peak intensities increased with increasing temperature. SiO_2_ crystalline phases including quartz present in the as-received coal fly ash were not nearly detected at 1400 °C. The presence of some silicate minerals might be expected but could not be clearly confirmed, due to their very weak peak intensities in relatively more glass melts. A secondary mullite phase, which formed in the system coal fly ash-Al_2_O_3_, was additionally identified in the diffraction patterns, (421), (002), (151), (122), (251) and (100). The XRD patterns for the specimens sintered at 1500 °C were very similar to those at 1400 °C and the diffraction intensity of mullite scarcely increased. Based on the XRD analysis, the formation of mullite in coal fly ash with additional α-Al_2_O_3_ is presumed as follows:

(I) prior to mullitization most SiO_2_ crystalline phases convert into SiO_2_-rich melts at temperatures higher than corresponding eutectic points in the Al_2_O_3_-SiO_2_ system containing various fluxing components, especially alkali metal oxides in coal fly ash; (II) the mullite formation begins by the reaction of α-Al_2_O_3_ and SiO_2_-rich melt at 1300 °C, and with increasing firing temperature, the formed mullite increases; (III) the mullitization reaction is nearly finished at 1400 °C.

Most minor oxide components in coal fly ash, especially including alkali components, play an important role in the formation of mullite because they can make low melting point liquids in the system Al_2_O_3_-SiO_2_; then, the amount of liquids formed will depend on the firing temperature. Phase relations in the ternary system X-Al_2_O_3_-SiO_2_ (X: minor oxide impurities in coal fly ash) have been reviewed [13]. For example, K_2_O and Na_2_O usually act as strong fluxing agents on Al_2_O_3_-SiO_2_ mixtures. The ternary eutectic of K_2_O-Al_2_O_3_-SiO_2_ decreases to 958 °C and Na_2_O exhibits a similar fluxing effect on K_2_O. The addition of even small amounts of Fe_2_O_3_ to Al_2_O_3_-SiO_2_forms a liquid phase. Even if each of Al_2_O_3_ (2054 °C), SiO_2_ (1726 °C), and CaO (2570 °C) has a high melting temperature, a liquid can form at temperatures lower than 1300 °C in the system CaO-Al_2_O_3_-SiO_2_. In addition, MnO has a strong fluxing effect on Al_2_O_3_-SiO_2_ mixtures with liquidus temperatures as low as ~1200 °C. Therefore, the richer the glass with low melting point components, the lower its melting point will become, and it will reduce the viscosity for any given temperature. This is accompanied by higher reaction rate for mullite formation via more rapid diffusion of the reaction species in more liquid phase.

**Figure 2:**
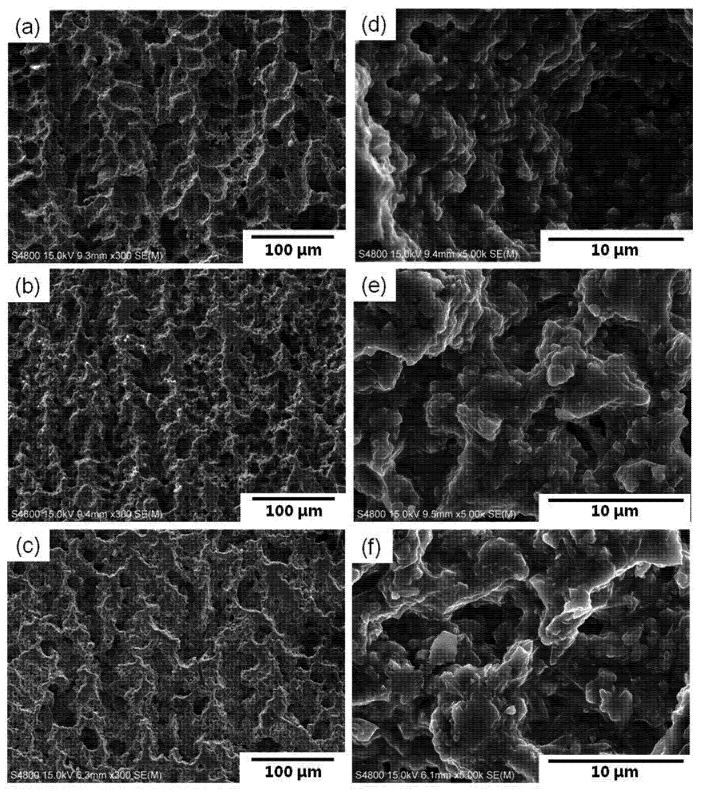
SEM images of cross-sections parallel to the macroscopic camphene ice growth direction; sintered at 1400 °C with (**a**,**d**) 30 wt.%; (**b**,**e**) 40 wt.% and (**c**,**f**) 50 wt. % solid loading, showing relatively dense walls (**d**,**e**,**f**).

Figure 2 shows SEM micrographs of cast bodies sintered at 1400 °C with solid loadings of 30–50 wt.%. Regardless of solid loading, similar dendrite-like microstructures were observed over a wide range, with the pores aligned along the camphene solid growth. At higher magnifications (Figure 2d,e,f), the walls surrounding the pores exhibited a relatively dense structure containing most granular-shaped agglomerates and a few rod-shaped mullite crystals. The stable mullite structure is orthorhombic, consisting of edge shared AlO_6_ octahedra oriented in the *c* direction and these octahedral chains are cross-linked by (Si, Al)O_4_ tetrahedra [14]. The approximate lattice constants are *a* = 0.755 nm, *b* = 0.769 nm and *c* = 0.288 nm (JCPDS card no. 15–776). Therefore, the crystal growth of mullite has a preferred orientation in the direction parallel to the *c* axis than in any other; in this case, the presence of increasing amounts of liquid phase especially at higher temperature can facilitate the formation and growth of rod-shaped mullite. Similarly, whisker-shaped mullite with an aspect ratio of >30 (0.6–1.8 μm in diameter) can be obtained from coal fly ash with the additions of NH_4_Al(SO_4_)_2_·12H_2_O and NaH_2_PO_4_·2H_2_O as alumina source and/or fluxing agents [15]. Hong *et al.* [16] obtained mullite grains with an aspect ratio of >10 and a length of >30 μm in the diphasic gel system (boehmite and silica) with the additions of 2 wt.% mullite whiskers and 2 wt.% B_2_O_3_, sintered at 1650 °C for 5 h. Such rod-shaped morphology was scarcely examined in cast bodies sintered at 1300 °C.

**Figure 3:**
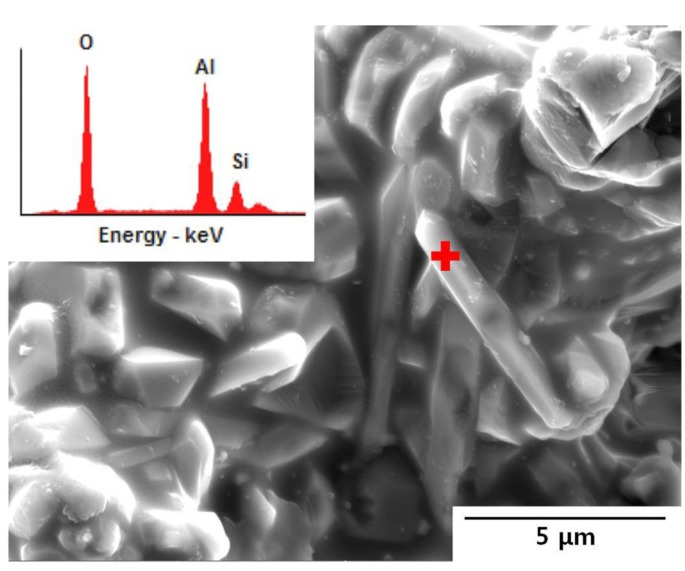
EDS spectrum on rod-shaped mullite formed in the cast body Al_2_O_3_/SiO_2_ = 2.0,molar ratio) of coal fly ash and alumina powders after sintering at 1500 °C for 2 h with 40 wt.% solid loading; this showing ion concentration (wt.%) of 43.40 O, 42.04 Al and 14.56 Si on a spot “+”.

The mullite structure theoretically can have any composition between sillimanite (*x* = 0.00) and aluminum oxide (*x* = 1.00) in the general formula Al_4+2*x*_Si_2−2*x*_O_10−*x*_ [17,18]. In this study, the average molar ratio of Al_2_O_3_/SiO_2_, determined by ED spectrum for fourteen rod-shaped mullite crystals (for example, as shown in Figure 3), existed in the walls of the cast bodies sintered at 1400 and 1500 °C was ~1.35, this corresponding to Al_2_O_3_-deficient mullite to stoichiometric composition (Al_2_O_3_/SiO_2_ = 1.5, molar ratio; *x* = 0.25). There is not the obvious reason why the compositions of the mullite crystals formed in the presence of a considerable amount of glass melt are different to each other and in most cases, limited to SiO_2_-rich mullite; however, the reason could be due to the limitations of the EDS beam analysis in the scanning electron microscopy and the crystal chemistry. Another possible reason could well be the high SiO_2_ content in the liquid phase formed on firing in the Al_2_O_3_-SiO_2_ system, compared with 3/2-mullite.

**Figure 4:**
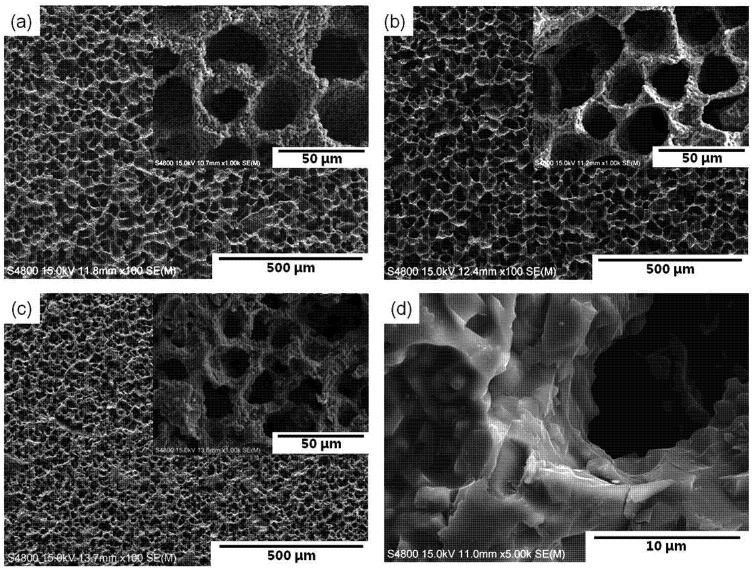
SEM images of cross-sections perpendicular to macroscopic camphene ice growth direction; sintered at (**a**) 1300 °C; (**b**) 1400°C;(**c**) 1500°C with 30 wt.% solidloading; and (**d**) sintered at 1500 °C with 50 wt.% solid loading.

**Figure 5:**
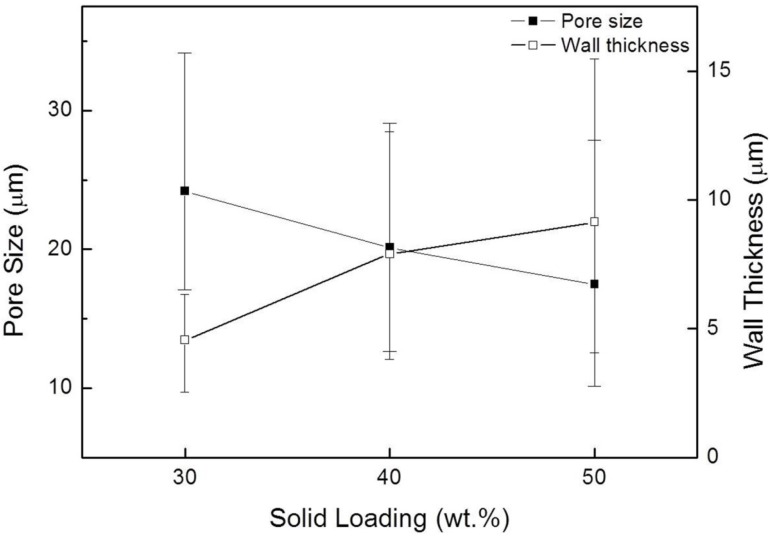
Pore size and wall thickness of porous mullite/alumina cast bodies sintered at 1400 °C as a function of solid loading; the error bars and square symbols indicate the maximum/minimum and average values, respectively.

Figure 4 shows SEM micrographs of the sintered microstructures at 1300–1500 °C developed with 30 wt.% solid loading and the microstructure sintered at 1500 °C with 50 wt.% solid loading. Unidirectional pore channels were observed in the cross-sections perpendicular to the solidification direction. With increasing sintering temperature, the average pore size decreased in the range 25.3–20.8 μm, possibly due to further firing shrinkage. In addition, the walls of the pore channels obtained from higher solid loading suspension showed a more densified microstructure containing inter-agglomerated granular and rod-shaped particles (Figure 4d). The pore size and wall thickness are also affected by solid loading, as shown in Figure 5. The average pore size (24.2–17.5 μm) was in inverse proportion to the wall thickness (4.6–9.1 μm). When compared with lower solid loading, the particle rejection at a freezing-front is less effective in higher solid loading suspension, this leading to the formation of smaller sized pores and thicker pore walls. In addition, the average pore size of the composite materials sintered at 1300–1500 °C was 20–25 μm at 30 wt.% solid loading, 18–20 μm at 40 wt.% and 15–17 μm at 50 wt.%; in such a case, the wall thickness was 3.5–6.7, 6.8–8.4 and 9.9–11.9 μm at 30, 40 and 50 wt.% solid loading, respectively. Relatively high deviation in both pore size and wall thickness appeared, presumably resulting from gradient solid concentration in suspension and/or inhomogeneous shrinkage of the interconnected porous structure during firing at a given temperature.

**Figure 6:**
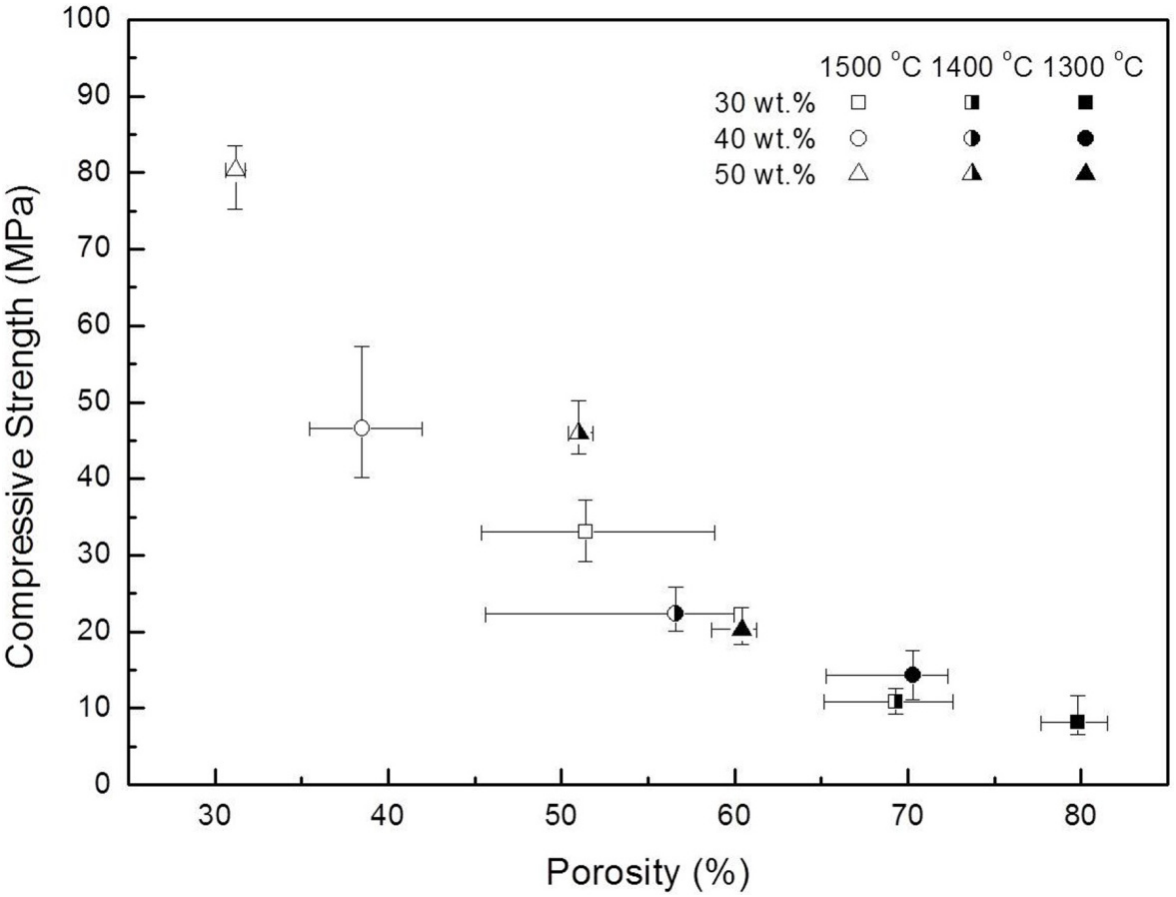
Porosity-compressive strength relationships of porous mullite/alumina composite processed with 30–50 wt.% solid loading; the error bars and square symbols indicate the maximum/minimum and average values, respectively.

The mechanical strength of porous ceramics is affected by several parameters, such as porosity, pore size distribution, pore shape, wall thickness and wall density, which are generally determined by production processes. However, it is difficult to consider all the parameters for evaluating the mechanical properties of porous ceramics since there are too many parameters that are interdependent and the mechanical properties are affected by all of them via the microstructure, especially in the case of sintered porous ceramics. Among these parameters, the degree of porosity greatly influences the mechanical strength of porous ceramics. In this study, therefore, the effects of initial solid loading and sintering temperature on the porosity and compressive strength have been examined and the compressive strength-porosity relationships are shown in Figure 6. The compressive strength generally increased with the reverse order of values for porosity, *i.e*., high porosity gave low compressive strength, due to the low bulk density of the sintered bodies. The porosity decreased with increasing temperature and the degree of porosity reduction was similar, regardless of solid loading. Consequently, the highest compressive strength (80.4 MPa) was obtained in the specimen with the lowest porosity of 31.2%, sintered at 1500 °C with 50 wt.% solid loading. On the other hand, the sintered body at 1300 °C with 30 wt.% solid loading exhibited the lowest compressive strength of 8.2MPa (79.8% in porosity). With increasing sintering temperature from 1350 to 1550 °C, the compressive strength of porous mullite/corundum ceramics produced by Yang *et al*. [19] increased from 3.1 to 11.7 MPa and porosity decreased from 81%to 78%.

The sintered mullite/alumina composites with unique pore structures and relatively dense walls obtained by this method should give high porosity, increased specific surface area, high fluid permeability and good mechanical strength, consequently satisfying requirements for various applications [4]. For instance, the resulting materials could be effectively used for waste-water treatment purposes, like the zeolitic materials prepared using CFB-derived coal fly ash [20].

## 3. Experimental Section

As-received coal fly ash (Samcheonpo Thermal Power Plant, Goseong, Korea) contained 54.20 SiO_2_, 19.42 Al_2_O_3_, 10.44 Fe_2_O_3_, 8.17 CaO, 1.64 MgO, 1.40 K_2_O, 1.74 TiO_2_, 1.33 SO_3_, 1.13 P_2_O_5_, 0.30 BaO, 0.15 SrO, 0.10 wt.% ZrO_2_ determined by X-ray fluoroscopy (PW2400, Philips, Chandler, AZ, USA) analysis and consisted mainly of silicate minerals with a specific surface area of 2.17 m^2^·g^−1^ and an agglomerate size of about 33.8 μm (<90%). Based on the results of XRF analysis, commercial Al_2_O_3_ powder (AES-11C, Sumitomo Chemicals, Tokyo, Japan) was added to the coal fly ash to adjust the molar ratio of Al_2_O_3_/SiO_2_ to 2.0. A mixture of coal fly ash and alumina powders was ball-milled in ethanol, rotary vacuum evaporated (Rotavapor R114, Buchi Co., Flawil, Switzerland), and then ground to pass through 200 mesh sieve. Reagent grade camphene (C_10_H_16_, Aldrich Chemistry, Milwaukee, WI, USA), Darvan-C (R.T. Vanderbilt Co., Norwalk, CT, USA), PVB (Aldrich Chemistry, Milwaukee, WI, USA) and Dynol 604 (Air Products and Chemicals, Allentown, PA, USA) were used as the freezing solvent, dispersant, binder and surfactant, respectively. Slurries with initial solid loadings of 30, 40 and 50 wt.% were prepared by putting a mixture of coal fly ash and alumina into warm liquid camphene containing appropriate amounts of processing additives. The mixed batches were ball-milled at 60 °C for 24 h and then de-aired by magnetically stirring under vacuum (WiseStir, Daihan Sci. Co., Wonju, Korea). The prepared warm slurries were poured into cylindrical polyethylene molds (30 mm diameter × 15 mm). After casting, the slurries were rapidly solidified from the direction of bottom using liquid nitrogen (Kyungdong Gas, Pusan, Korea). After demolding, the frozen samples were placed in a freeze dryer (TFD 5505, IlShinBioBase Ltd., Dongduchun, Korea) to sublimate the camphene ice. After calcining at 600 °C to remove organic additives, the green compacts were sintered at 1300–1500 °C (heating rate of 3 °C·min^−1^) for 2 h in air.

The sintered bulk density and porosity was measured using the Archimedes principle. The crystalline phases were determined by X-ray diffractometry (XRD, D-MAX II, Rigaku, Tokyo, Japan). Microstructures of the sintered specimens were examined with scanning electron microscopy (SEM, JSM-500, Jeol, Tokyo, Japan). The compressive strength was measured in the direction parallel to the macroscopic freezing direction for five sintered specimens (20 mm diameter × 10 mm height) using an Universal Testing Machine (Model 6025, Instron, Norwood, MA, USA) with a cross-head speed of 1.0 mm·min^−1^ and a load cell of 1 kN.

## 4. Conclusions

Porous mullite-alumina composites with uniform dendrite-type morphology were produced by the camphene-based freeze casting of coal fly ash/alumina mixed powders; in this route, the pore channels with circular-like cross-sections were also preferentially oriented along the direction of freezing. Both initial solid loading and sintering temperature greatly altered the resulting porosity, pore size, and pore wall thickness, consequently determining the compressive strength of porous bodies. By increasing solid concentration (30–50 wt.%) and sintering temperature (1300–1500 °C), the porosity (79.8%–31.2%) and pore size (25–15 μm) decreased but the wall thickness (3.5–11.9 μm) increased. The compressive strength increased from the reverse order of values for porosity. The average compressive strength increased from 8.2 MPa to 80.4 MPa, decreasing the porosity from 79.8% to 31.2%. Consequently, this processing technique for recycling of coal fly ash to fabricate porous ceramic composites with controlled porosity and desirable compressive strength appears to be very effective; moreover, since the compressive strength behaves in an opposite manner to the porosity, it is a relatively easy to select the optimum processing conditions for obtaining a specific requirement for any particular engineering application.

## References

[1] Jeong H.S. (2013). Coal Ash as Resources. Electimes.

[2] Schneider H., Schreuer J., Hildmann B. (2008). Structure and properties of mullite—A review. J. Eur. Ceram. Soc..

[3] Gibson L.J., Ashby M.F. (1999). Cellular Solids Structure and Properties.

[4] Ishizaki K., Komarneni S., Nanko M. (1998). Porous Materials Process Technology and Applications.

[5] Minh N.Q. (1993). Ceramics fuel cells. J. Am. Ceram. Soc..

[6] Studart A.R., Gonzenbach U.T., Tervoort E., Gauckler L.J. (2006). Processing routes to macroporous ceramics: A review. J. Am. Ceram. Soc..

[7] Deville S. (2010). Freeze-casting of porous biomaterials: Structure, properties and opportunities. Materials.

[8] Li W.L., Lu K., Walz J.Y. (2012). Freeze casting of porous materials: review of critical factors in microstructure evolution. Inter. Mater. Rev.

[9] Deville S., Saiz E., Nalla R.K., Tomsia A.P. (2006). Freezing as a path to build complex composites. Science.

[10] Macchetta A., Turner I.G., Bowen C.R. (2009). Fabrication of HA/TCP scaffolds with a graded and porous structure using a camphene-based freeze-casting method. Acta Biomater.

[11] Chen R., Wang C.A., Huang Y., Ma L., Lin W. (2007). Ceramics with special porous structures fabricated by freeze-gelcasting: Using tert-butyl alcohol as a template. J. Am. Ceram. Soc..

[12] Koh Y.H., Lee E.J., Yoon B.H., Song J.H., Kim H.E. (2006). Effect of polystyrene addition on freeze casting of ceramic/camphene slurry for ultra-high porosity ceramics with aligned pore channels. J. Am. Ceram. Soc..

[13] Schneider H., Okada K., Pask J. (1994). Mullite and Mullite Ceramics.

[14] Jaaski J.Y., Nissen H.U. (1983). Investigation of superstructures in mullite by high resolution microscopy and electron diffraction. Phys. Chem. Miner..

[15] Park Y.M., Yang T.Y., Yoon S.Y., Stevens R., Park H.C. (2007). Mullite whiskers derived from coal fly ash. Mater. Sci. Eng. A.

[16] Hong S.H., Germignani W., Messing G.L. (1996). Anisotropic grain growth in seeded and B_2_O_3_-doped diphasic mullite gels. J. Eur. Ceram. Soc..

[17] Burnham C.W. (1964). Crystal structure of mullite. Carnegie Inst. Washington Yearb..

[18] Burnham C.W. (1964). Composition limits of mullite and the sillimanite-mullite solid solution problem. Carnegie Inst. Washington Yearb..

[19] Yang F., Li C., Lin Y., Wang C.A. (2012). Effects of sintering temperature on properties of porous mullite/corundum ceramics. Mater. Lett..

[20] Koukouzas N., Vasilatos C., Itskos G., Mitsis I., Moutsatsou A. (2010). Removal of heavy metals from wastewater using CFB-coal fly ash zeolitic materials. J. Hazard. Mater..

